# The varied experience of undergraduate students during the transition to mandatory online chem lab during the initial lockdown of the COVID-19 pandemic

**DOI:** 10.1186/s43031-022-00055-0

**Published:** 2022-04-13

**Authors:** Joseph Watts, Kent J. Crippen, Corey Payne, Lorelie Imperial, Melanie Veige

**Affiliations:** University of Florida, School of Teaching and Learning, PO Box 117048, Gainesville, FL 32611 USA

**Keywords:** Phenomenography, Online Laboratory Education, Undergraduate, Transition, Pandemic

## Abstract

The radical global shift to online teaching that resulted from the initial lockdown of the COVID-19 pandemic forced many science educators into the predicament of translating courses, including teaching laboratories, that were based upon face-to-face or practical goals and conventions into ones that could be delivered online. We used this phenomenon at the scale of a research-intensive, land-grant public institution to understand the various ways that the switch was experienced by a large cohort of 702 undergraduate students taking General Chemistry Laboratory. Data was collected over 3 weeks with identical surveys involving four prompts for open-ended responses. Analysis involved sequential explanatory mixed methods where topic modeling, a machine learning technique, was used to identify 21 topics. As categories of experience, these topics were defined and further delineated into 52 dimensions by inductive coding with constant comparison. Reported strengths and positive implications tie predominantly to the topics of Time Management Across a Lab Activity and a Critique of Instruction. Consistent with other reports of teaching and learning during the pandemic, participants perceived Availability of the Teaching Assistant for Help as a positive implication. Perceptions of weakness were most associated with Having to Work Individually, the Hands On Experience, a Critique of Instruction, and Learning by Doing. Hands on Experience, which was interpreted as the lack thereof, was the only topic made up nearly entirely of weaknesses and negative implications. The topic of Learning by Doing was the topic of greatest occurrence, but was equally indicated as strengths, positive implication, weakness, and negative implication. Ramifications are drawn from the weaknesses indicated by students who identified as members of an underrepresented ethnic minority. The results serve as a reminder that the student experience must be the primary consideration for any educational endeavor and needs to continue as a principal point of emphasis for research and development for online science environments.

## Introduction

The radical global shift to online teaching and learning that resulted from the lockdown rules and regulations of the COVID-19 pandemic (henceforth, pandemic) in the spring of 2020 forced many science educators into the predicament of translating courses that were based upon face-to-face or practical goals and conventions into ones that could be delivered online (Gewin, 2020). This included experiential courses such as teaching laboratories in science and engineering, for which many had never been intended nor developed to be offered online. According to the national undergraduate survey in the United States (U. S.) by Means et al., (2020), the general response of colleges and universities was to enter a *triage mode*, moving postsecondary courses online with little time to contemplate research-based practice and equity concerns (p. 3). The goal of this study is to offer a first-person perspective on this phenomenon from 702 undergraduate students who were enrolled in *General Chemistry Laboratory* at a research-intensive, land-grant public institution in the U. S.

For the last few decades, in the pursuit of improving how students act upon the world, student experience has been a major topic of interest within science education (Millar, 2004). Research consistently shows that a learner’s prior experience has a strong influence on how they come to understand a topic (Kalyuga, 2007; Simonsmeier et al., 2021). Indeed, students’ perception of science is influenced by a growing number of influences that include self-efficacy, socio-cultural factors, interest, school guidance, gender, and so on. In addition, the advancement of technology, including its use for informal learning or more formally for educational purposes, has led to students approaching learning differently (Thompson, 2013). While knowledge and understanding are core outcomes that follow from a science education experience, discerning the various sentiments of diverse participants related to a scientific journey are equally important (National Research Council [NRC], 2006). This implies a need for exploring the range of what might be possible for a given learning context, including the elements or attributes of the environment that learners deem critical for success.

Learning science via laboratory, which is known in some contexts as practical work and will be referenced here as laboratory education, is recognized as an essential part of university science education (Lunnetta et al., 2007), where students follow procedures, perform experiments, and demonstrate skills and knowledge of associated concepts. Formal courses of this genre, which typically occur in specially designated teaching laboratories, have become an increasingly promising subject of examination due to the inherent connection to the inquiry process and more authentic forms of professional practice (de Jong et al., 2013; Reid & Shah, 2007). However, the expansion of online education has spawned an environment rife with challenges that must be overcome in order to maintain these goals, which are largely tied to historical assumptions and the affordances of face-to-face experiences (Means et al., 2020), such as the necessity for dangerous materials or costly equipment (Nolen & Koretsky, 2018).

The switch from an on-campus, physical resource-dependent and experience-driven model to a form that could be delivered and supported entirely over the Internet at a massive scale occurred in a matter of days in the spring of 2020 and had a drastic effect upon everyone involved. Applying the theoretical framework of phenomenography, we used this situation at the scale of a research-intensive, land-grant public institution as an opportunity to understand the various ways that the switch to online learning was experienced by undergraduate students taking General Chemistry Laboratory. In doing so, we sought to better understand the general barriers to participation as well as more specifically, those for achieving the general intent of laboratory education. The lessons learned offer a unique and unparalleled opportunity to examine our assumptions about laboratory education as well as for supporting the needs and interests of all students. Accordingly, we sought to address the following research questions through a case study of our context: 1) What different ways did students perceive their experience with a required transition to a mandatory online chemistry laboratory during the COVID-19 pandemic? 2) In what ways did the experience of students who identified as members of an underrepresented ethnic minority (URM) differ from that of their peers? The case was bounded by our intent to understand the full variation in experiences and the ways of seeing and understanding (Yates et al., 2012) during the government mandated transition from in-person to online learning that occurred in response to the pandemic.

### Theoretical framework

Phenomenography is used as a theoretical framework to address questions pertaining to thinking and learning by evaluating the variation in experience with a particular phenomenon (Marton & Booth, 1997). In order to document this variation, assumptions are limited to the nature of participants’ reality, emphasizing the way people experience the phenomenon both in relation to and being distinguished from its parts. Prior experience informs new perceptions and results in different interpretations of the same experience (Han & Ellis, 2019).

The variation in understanding by a group of learners is limited by the number of qualitatively unique ways a particular phenomenon is conceptualized (Marton, 1981, 1992). Phenomenographic researchers assume the existence of a finite number of ways to understand, perceive, or experience a phenomenon of interest (Tight, 2018). This clarification is necessary as a comparison of definable relationships is essential in a learning context (Boda, 2019). If drawn from a representative sample, such relationships can permit meaningful arguments of conceptions given a similar context (Feldon & Tofel-Grehl, 2018; Marton & Booth, 2013).

As a second-order approach that gives priority to how participants see and understand the world, phenomenography offers insight into students’ current and evolving conceptual understanding. Science education researchers typically use phenomenography to inform pedagogy and curriculum with the intent of positively impacting student learning. Phenomenography is useful in the identification of variation in student experience to examine learning disparities (Newton & Martin, 2013). This is especially the case in mixed methods research where the validity of idiographic understandings must be balanced with the nomothetic insights that occur within scientific inquiry (Feldon & Tofel-Grehl, 2018). Phenomenographic exploration increases in value in parallel to trends in practice that can outpace learners. As such, phenomenography serves as a viable framework to efficiently engage the spectrum of effects following the rapid technology integration during the government mandated transition from in-person to online learning that occurred in response to the pandemic.

### Review of related literature

Regardless of the educational level, laboratory education emphasizes scientific practice through the use of inquiry strategies as a core component (Gott & Duggan, 1996). Barriers to implementation of inquiry in laboratory education have been omnipresent due to instructor beliefs surrounding the nature of science itself (Crawford, 2007). Regardless, research has shown that the inclusion of laboratory education improves student learning outcomes compared to those in a lecture-only curriculum (Merchant et al., 2012). Educators of all levels often rely on a lecture paradigm with a textbook as the instructional core (Linn & Eylon, 2011). Regardless of the educational level, supplementing traditional lessons with hands-on investigations accounts for around 50% of the total lesson, a proportion which decreases further when instructors utilize computer technology (Linn & Eylon, 2011). While national guidelines are lacking, the majority of undergraduate science and engineering majors emphasize the grasp of laboratory concepts as mandatory for a degree (Reid & Shah, 2007).

Successful laboratory education strives to emulate the conditions and thought processes of practicing researchers, using critical thinking to translate arguments beyond the basic knowledge of how to complete content specific tasks (Wan et al., 2020). While alternative computer-based laboratory activities have successfully been implemented in curricula by some instructors, these activities have tended to function as an appurtenance to in-person laboratory experiences rather than a functional replacement (Rowe et al., 2017). Additionally, comparing the effectiveness of online education against in-person laboratory education is complicated due to historically small sample sizes and insufficient standardized educational objectives (Ma & Nickerson, 2006; Rowe et al., 2017). For example, the corpus of research on laboratory activities in virtual reality are largely comparison studies for knowledge acquisition where it is assumed that a virtual version provides a more accessible emulation of a physical alternative (Reeves & Crippen, 2020). Notably, a significant change in education in recent years involves how information and communication technologies and other digital tools have altered information sharing and processing speeds (Fraillon et al., 2014).

While digital environments can enhance science learning, in practice, researchers must also be wary of the potential for broadening cultural and socio-economic gaps in access and participation (Bolaños & Salinas, 2021). Inequalities have been further escalated during the pandemic, with new family responsibilities, expanded need for Internet access, general living conditions, and financial restrictions being added to the list of education-related problems (Engelbrecht et al., 2020). Instructors have also been forced to rapidly adapt their teaching approaches to a virtual environment, a practice some have referred to as *Panic-gogy* (Kamanetz, 2020). The variation in computer technology that schools have access to also plays a part in the potential for a digital divide. While data are lacking in the U.S., recent research suggests that a positive correlation exists between teacher access to information and computer technologies (ICT) and successful adaptation to an online curriculum (König et al., 2020). While early-career instructors may be more tech-savvy, their digital skills are not enough to compensate for an institution’s lag in ICT transformation processes.

Active engagement is paramount to the learning process and mandatory for grasping the nature of science from a general chemistry laboratory. Online laboratory experiences offer a unique combination of advantages and weaknesses (Potkonjak et al., 2016). Research on virtual laboratory experiences has shown an enhancement in performance and learning due to students’ ability to practice essential concepts in a time efficient and safe space before entering a physical laboratory (Wang et al., 2014). While online laboratory experiences are effective preparation tools, they lack the inherent characteristics and tactile information that physical experiences offer (de Jong et al., 2013). Despite the differences between online and physical laboratory experiences, some researchers have shown promising results in the support of online laboratory education as an equal alternative to face-to-face methods (Makransky et al., 2016). To enhance learning in the online setting, laboratory education coordinators are advised to develop a virtual laboratory system complete with a full software integration rather than opt for a far more complicated physical laboratory with remote access. At minimum, online chemistry laboratory education should be complete with observations, hypotheses, and other experimental designs.

Despite the challenges associated with the pandemic, students’ progression through general chemistry laboratory and other STEM courses has changed with the increasing necessity of online transitions. Virtual engagements are not uniform and vary based on reliable Internet connections and access to technology to properly access data, with such issues vocalized by students in the present study (Brenner et al., 2021).

### Methodology

**Fig. 1:**
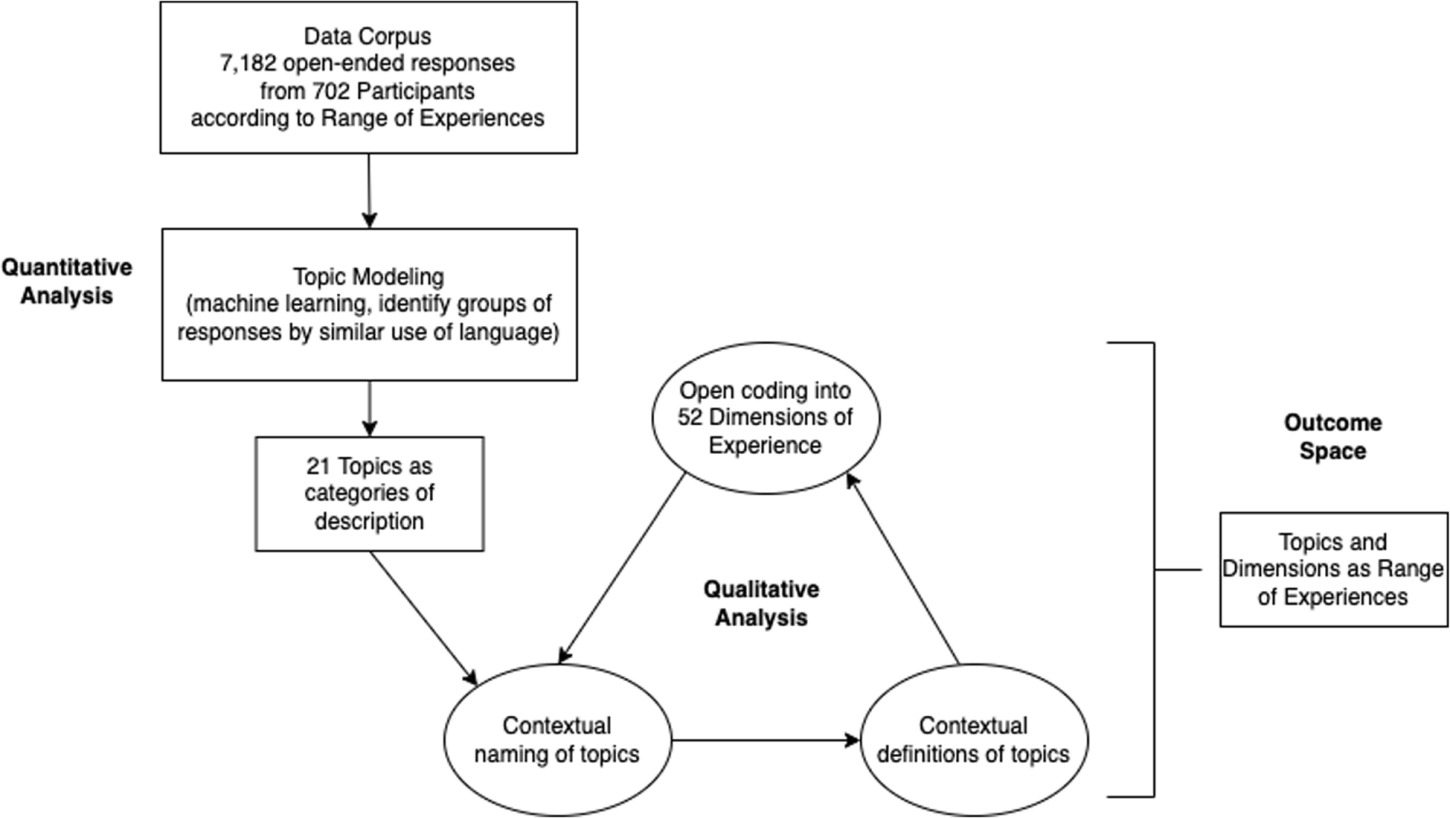
The process of our sequential explanatory mixed method

This study used a sequential explanatory mixed methodology to investigate the experiences of undergraduate students taking General Chemistry Laboratory during the spring of 2020 at a university in the southeastern U. S. (Creswell & Clark, 2017). The quantitative-to-qualitative analysis pathway, which resulted in identification of an outcome space, is visualized in Fig. 1. Analytical topic derivation is exemplified in Table 3.

### Context

The context for this study was a first-semester general chemistry laboratory course, which coincided with a lecture course. These are service courses that support a range of STEM majors and students ideally take both courses during the same semester and the laboratory activities are designed to match the content of the lecture course. The laboratory course is facilitated by a cadre of teaching assistants (TAs), typically thirty or more, with a TA to student ratio of roughly 1:16. Each TA meets with the same collection of students once per week. Students attend one three-hour laboratory period per week (ten unique meetings per semester) and work with a randomly assigned partner. During the 2020 Spring semester, the course began on January 6 and progressed on schedule until March 13 where the final four laboratory activities operated online asynchronously.

The course content involves practical experiments related to chemistry, as well as experiences with common laboratory practices, techniques, and experimental processes (Table 1). Students use a variety of instruments for hands-on activities, such as volumetric glassware, balances, Bunsen burners, micropipettes, redox and conductivity probes, a spectrophotometer, temperature probes, and an assortment of chemicals. They make observations of rates of bleaching of dyes, single and double displacement reactions, build physical molecular models, and learn graphing techniques in spreadsheets. The course is supported by the campus learning management system and concludes with a cumulative written examination.

**Table 1:** Overview of the laboratory experiences for the course

Meeting Number	Dates	Content Theme	Activity Description	Format
1	January 20–24	Density	Measurements, use of volumetric glassware, accuracy, precision, introduction to Excel (generation of calibration curve, trendlines), determination of an unknown concentration of a solution based on density, and density determination of a solid.	Physical, Face-to-Face
2	January 27–31	Hydrates	Use of a Bunsen burner, safe handling of hot objects. Identification of hydrates and determining percent error; error analysis. Safety focus: safe handling of hot objects, use of Bunsen burner.	Physical, Face-to-Face
3	February 3–7	Stoichiometry	Introduction to micropipettes. Redox titration using ORP probe; rough titration and fine titration. Interpretation of graphs. Advanced graphing in Excel (formulas for first and second derivative curves). Error analysis.	Physical, Face-to-Face
4	February 10–14	Gases	Collection of a gas over water, reaction stoichiometry, reading a barometer, error analysis. Safety focus: interpreting an SDS.	Physical, Face-to-Face
5	February 17–21	Calorimetry	Experimental determination of heat of solution, heat of neutralization, heat of reaction, and of specific heat of a metal using coffee cup calorimetry. Error analysis. Safety focus: hazards and risks.	Physical, Face-to-Face
6	February 24–28	Dilution and Beer’s Law	UV-VIS spectroscopy, serial dilution, generation of calibration curve of absorbance vs concentration, determination of concentration of an unknown. Graphing and error analysis. Safety focus: pictograms on SDS.	Physical, Face-to-Face
7	March 2–6	None	Spring Break	
8	March 9–13	None	Preparation for mandatory online	
9	March 16–20	Reaction Kinetics	Qualitative and quantitative exploration of reaction kinetics. What factors affect the rate of a reaction (qualitative), determination of the rate constant for a reaction using graphical techniques (quantitative). Graphing and error analysis. Reproducible use of micropipette and UV-VIS. Safety focus: CAS registry numbers, definitions of key safety terms.	Mandatory Online
10	March 23–27	Ions and Electrolytes	Flame tests of metal ions, energy/wavelength calculations, discussion of electronic transitions. Use of a conductivity probe to measure conductivity of solutions; discussion of what factors affect conductivity. Preparation and observation of microscale single- and double-displacement reactions, net ionic equations, classification of type of reaction. Safety focus: the NFPA diamond; differentiating between flammability and combustibility; definitions of key terms.	Mandatory Online
11	March 30–April 3	Lewis Structures	Lewis structures, wedge and dash 3D representations of molecules, polarity, building 3D models. Safety focus: types of goggles.	Mandatory Online
12	April 6–10	None	Make-up days for the staggered start to the semester.	Mandatory Online
13	April 13-17	Colligative Properties	Use of a temperature probe. Determination of boiling point elevation and freezing point depression. Determination of an unknown based on freezing point. Van’t Hoff factors. Graphical analysis and error analysis.	Mandatory Online

Lab exam 4/9: multiple choice. Safety, conceptual problems, calculations, making inferences

Spring Break 2020 was scheduled for March 2–6 and following the state government’s decision to lock down, students were instructed to return home for the remainder of the semester and that following the break, courses would only be available online. Following 2 weeks of work by the faculty, staff and TAs, on March 13, 2020 the course transitioned to asynchronous online delivery for the last 4 weeks of the activities. As part of the transition, new student resources were created, which included videos of laboratory staff performing procedures, optional synchronous sessions with TAs, and data sets for videotaped experiments that students were to analyze. Due to the time crunch in getting materials prepared, the videos did have any audio or captions. TAs offered optional office hours/help sessions during what was the scheduled meeting time in the institution’s time zone. Students worked individually on one activity per week using information provided via the learning management system. Students were spread across the country and world, so no synchronous sessions were required.

### Participants

Participants were undergraduate students enrolled in the general chemistry laboratory course and pursuing various majors. All students in the course were invited to participate and 702 (70.1%) became participants after providing informed consent. Participants were 85.7% freshmen, 65.6% identified as female, and 24.2% identifying as either: African American, American Indian/Alaskan Native or Hispanic, which we defined as URM based upon the criteria used by the U. S. National Science Foundation (Table 2). Given the prerequisite requirements for the course, we assumed that all students had some degree of prior knowledge for the concepts in the laboratory activities and potentially for the activities themselves.

**Table 2:** Summary of participant demographics

Characteristics	N	%
Ethnicity		
American Indian or Alaska Native	1	0.1%
Asian	119	17.0%
Black or African American	40	5.7%
Hispanic or Latin(x)	129	18.4%
Native Hawaiian or other Pacific Islander	2	0.3%
Multiracial	4	0.6%
White	391	55.7%
Prefer not to say	16	2.3%
URM		
Gender		
Female	108	62.1%
Male	64	36.8%
Non-binary/3rd gender	0	0.0%
Prefer to self-describe as something else	1	0.6%
Prefer not to say	1	0.6%
Major		
Science	58	33.3%
Engineering	41	23.6%
Health & Psychology	42	24.1%
Other	33	19.0%
non-URM		
Gender		
Female	342	66.8%
Male	162	31.6%
Non-binary/3rd gender	4	0.8%
Prefer to self-describe as something else	0	0.0%
Prefer not to say	4	0.8%
Major		
Science	186	106.9%
Engineering	118	67.8%
Health & Psychology	130	74.7%
Other	78	44.8%

### Data collection and analysis

Following the transition, three identical weekly surveys with four open-ended response prompts were administered (4/2, 4/6, 4/18). Open-ended questions are a recognized and efficient way of obtaining a range of experiences (Ashworth & Lucas, 2000). In an effort to best capture the full range of experience, including both positive and negative as well as affordances and barriers (Han & Ellis, 2019), these surveys asked students to “please use your experience *in the last week of this course* as your frame of reference.” and then included prompts for their views of the strengths (“What do you view as the *strengths* of this week’s experience with online chemistry laboratory?”, weaknesses (“What do you view as any *weaknesses* of the experience?”), new opportunities or positive implications (“What would you say are *new opportunities or positive implications* that have been afforded to you by this experience?”) and potential short- or long-term negative implications (“What do you view as any *potential short- or long-term negative implications* of the experience?”). Each response was recorded as an open-ended text response. The survey prompts were exactly the same for each week and students were required to respond in some fashion, even if to report *none*. Participant demographics were captured with a series of four closed-response items that appeared after the open-response items on the first survey that queried academic standing (e.g., freshman, sophomore, etc.), gender, major and ethnicity.

The content of the 7182 survey item responses were subjected to topic modeling (Nikolenko et al., 2017) using the Gibbs sampling Dirichlet mixture model as part of version 8.1.0 of the Text Processor extension in the application RapidMiner (Kotu & Deshpande, 2015). Topic modeling is a machine learning technique based upon natural language processing that assumes that each response, in this case, consists of exactly one topic. Data analysis under Latent Dirichlet Allocation (LDA) assumes that coding follows a distribution of categories (topics) prior to delineation of dimensions. Higher numbers of dimensions are preferable to distinguish words with inverse meanings. Data pre-processing involved a removal of terms by stemming, then removal of numbers, as well as stop and very short words. Using the maximum log likelihood optimization method (Sbalchiero & Eder, 2020), we determined the number of detectable topics to be 21. One topic was assigned to each response in the dataset, but we also acknowledge that a response may have contained multiple topics and thus additional coding would be required to check the validity of topic modeling. While such instances were apparent during the coding process, multiple sets of topics can be connected to determine potential dependencies cross-field through inclusion of correlated topic models (Blei & Lafferty, 2007; Salomatin et al., 2009). The steps used in the analytical derivation of Topic 11, which was ultimately named *Preparation for Future Laboratory Work* as part of the qualitative analysis, is provided as an example in Table 3. We operated under the assumption that these topics represented the principal categories of description and our use of machine learning not only afforded our capacity for working with a large dataset, but it allowed presuppositions to be set aside (i.e., bracketing) (Ashworth & Lucas, 2000).

**Table 3:** The analytical derivation of Topic 11 as Preparation for Future Laboratory Work using sequential explanatory mixed methods

Step	Description	Result	Source
1	Topic Number	11	Topic Modeling
2	Key words and Number of Occurrences	lab (468), futur (176), experi (174), equip (131), chemistri (118), skill (113), cours (108), laboratori (90), practic (75), student (71), chem (69), knowledg (67), class (61), lack (51), tool (51), level (45), set (44), know (43), semest (42), handl (42)	Topic Modeling
3	Example Responses(date, participant number, prompt)	“The strengths of this week’s experience would be gaining additional knowledge on bonding and resonance structures.” (4/6, 9024, Strength) “I feel that since I was not able to use the tools necessary for the laboratory myself, I am not familiar with them and will not be comfortable using them in future upper-level laboratory classes.” (4/2, 1191, Weakness) “With this lab being offered online, I am able to continue practicing the mathematics necessary for future chemistry activities, lessons, assignments, etc.” (4/18, 2790, Opportunity) “My practical lab skills are not able to be improved via an online format.” (4/23, 10,644, Negative Implication)	Topic Modeling
4	Construct Topic Name	Preparation for Future Laboratory Work	Qualitative Analysis^a^
5	Construct Topic Definition	Concerns about preparedness for future laboratory work in relation to other courses or for their career intentions.	Qualitative Analysis^a^
6	Identify and Code Topic Dimensions	Lack of hands-on experience (67^b^) Not prepared for future courses (59^b^) Lack of Equipment knowledge/skills (24^b^) Less experience in a lab (15^b^)	Qualitative Analysis^a^

^a^Recursive constant comparison

^b^Number of occurrences

Subsequent qualitative analysis was completed using constant comparison and discussion to consensus in order to construct contextual names and definitions for the topics as well as to identify dimensions of each (Yin, 2002). Qualitative analysis was completed by the first four authors. The de-identified responses, grouped by prompt and without inclusion of demographic information, were used for thematic coding and construction of the topic names and definitions were completed through iterative cycles of analysis, reflection and discussion.

Participant responses were first read across all categories in order to develop a sensitivity to the conceptions being described (Ashworth & Lucas, 2000). Following this reading, initial names and definitions were written for each topic. In a search for variation and meaning (Yates et al., 2012), this step was followed by a systematic open coding of responses as dimensions of the experience where each coder was responsible for one type of prompt (i.e., strength, weakness, etc.). While all responses were coded inductively, responses were coded in small batches with discussion and consensus after each that focused on integration and consistency across the types of prompts. Fifty-two dimensions were identified with names such as Adaptability (own pace, time, efficiency), Accommodating (communication, availability, help)*,* Videos, and Independence and Self-Reliance. These discussion and consensus sessions also resulted in revisions to the topic names and definitions. For example, Topic 7 began as *Expectations for Collaborative Wor*k, but became *Having to Work Individually* as the primary dimensions of Lack of Collaboration (e.g., “A weakness of the experience was not having a partner to discuss the process with.”), Lack of a Teaching Assistant (e.g., “hard to understand without TA”), and Lack of Instruction (e.g., “The weakness of this experience was having to find everything that we needed on your own.”) were delineated.

**Fig. 2:**
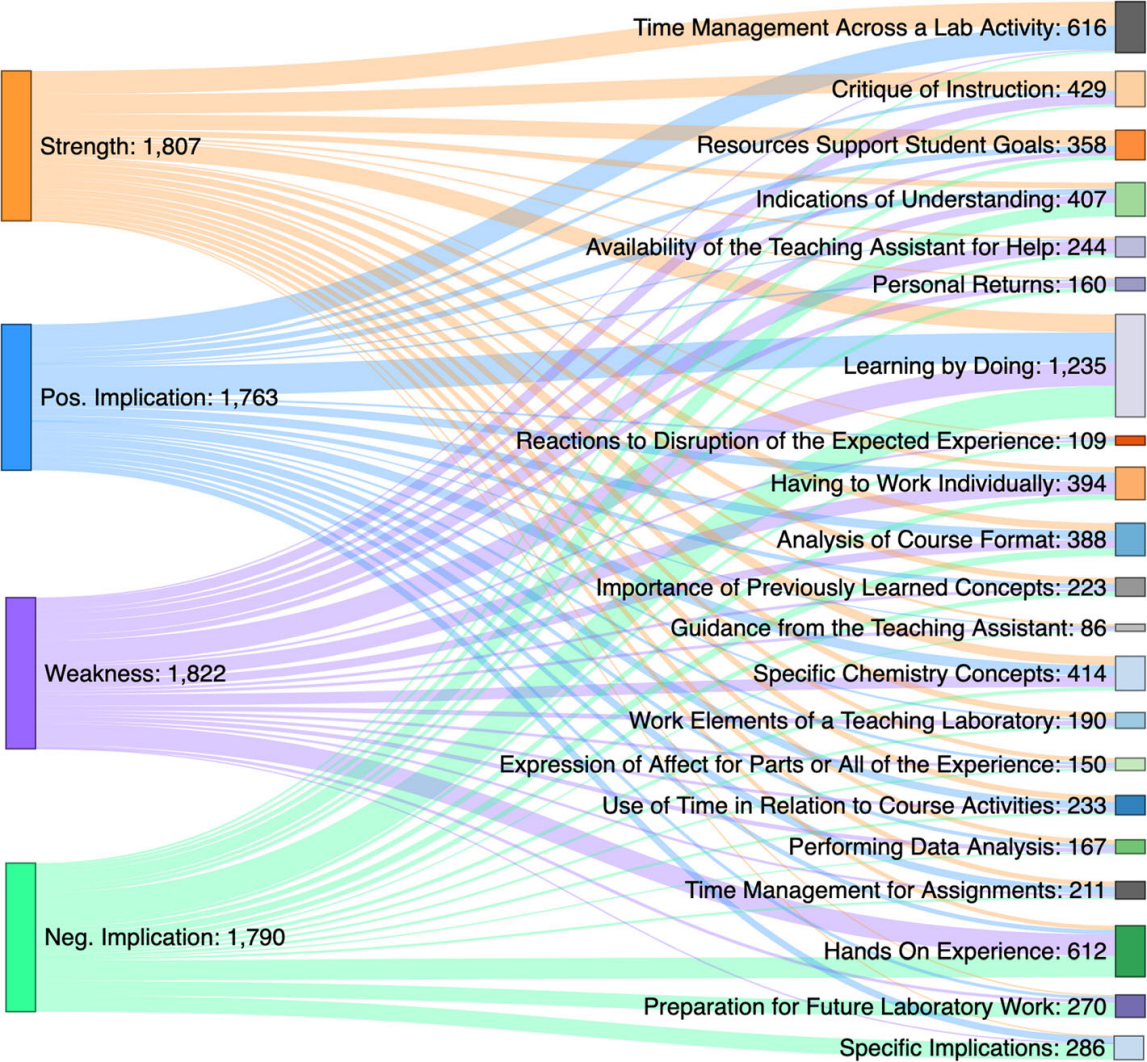
Topic response by prompt for all students

**Fig. 3:**
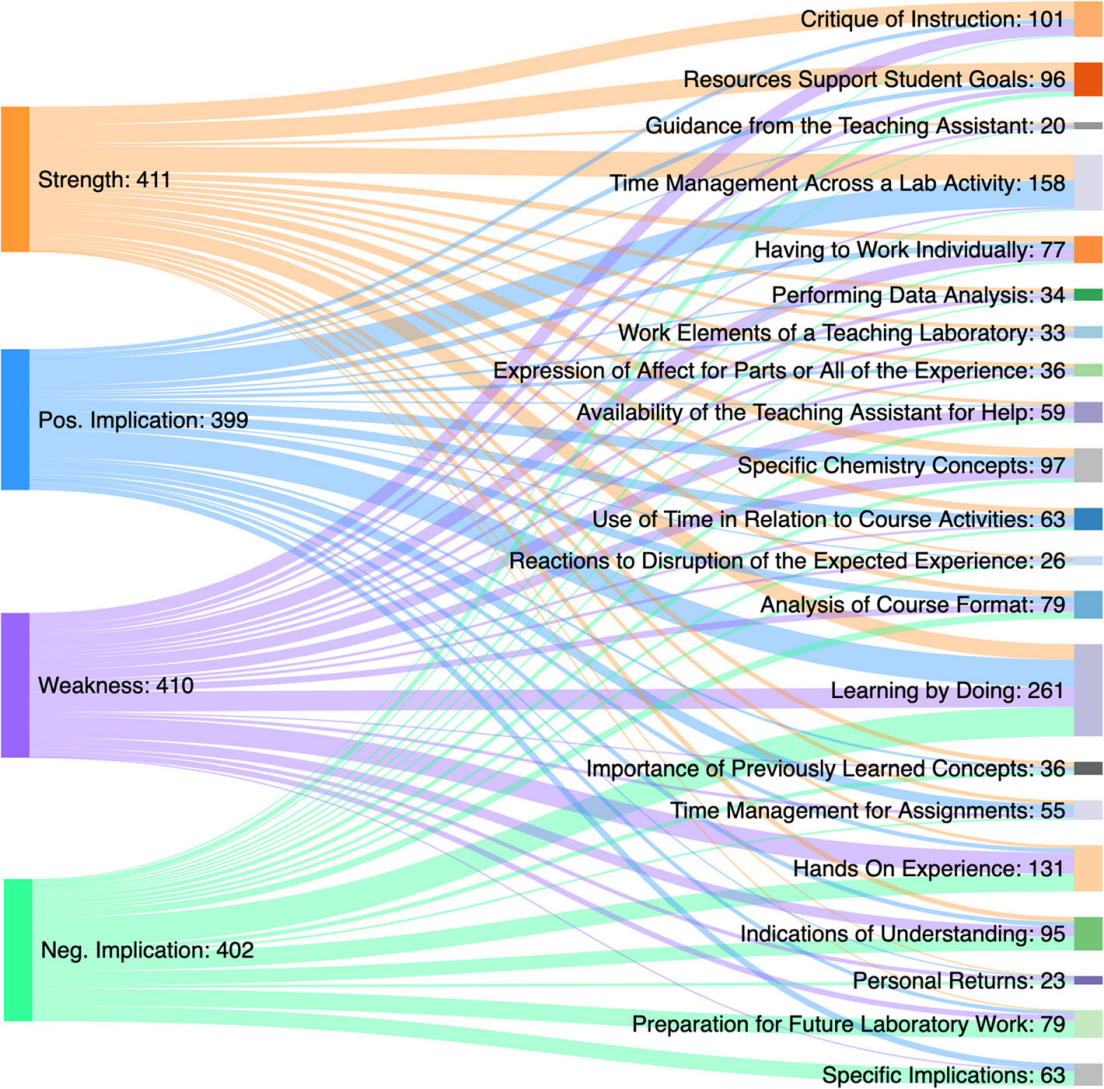
Topic response by prompt for URM students only

**Fig. 4:**
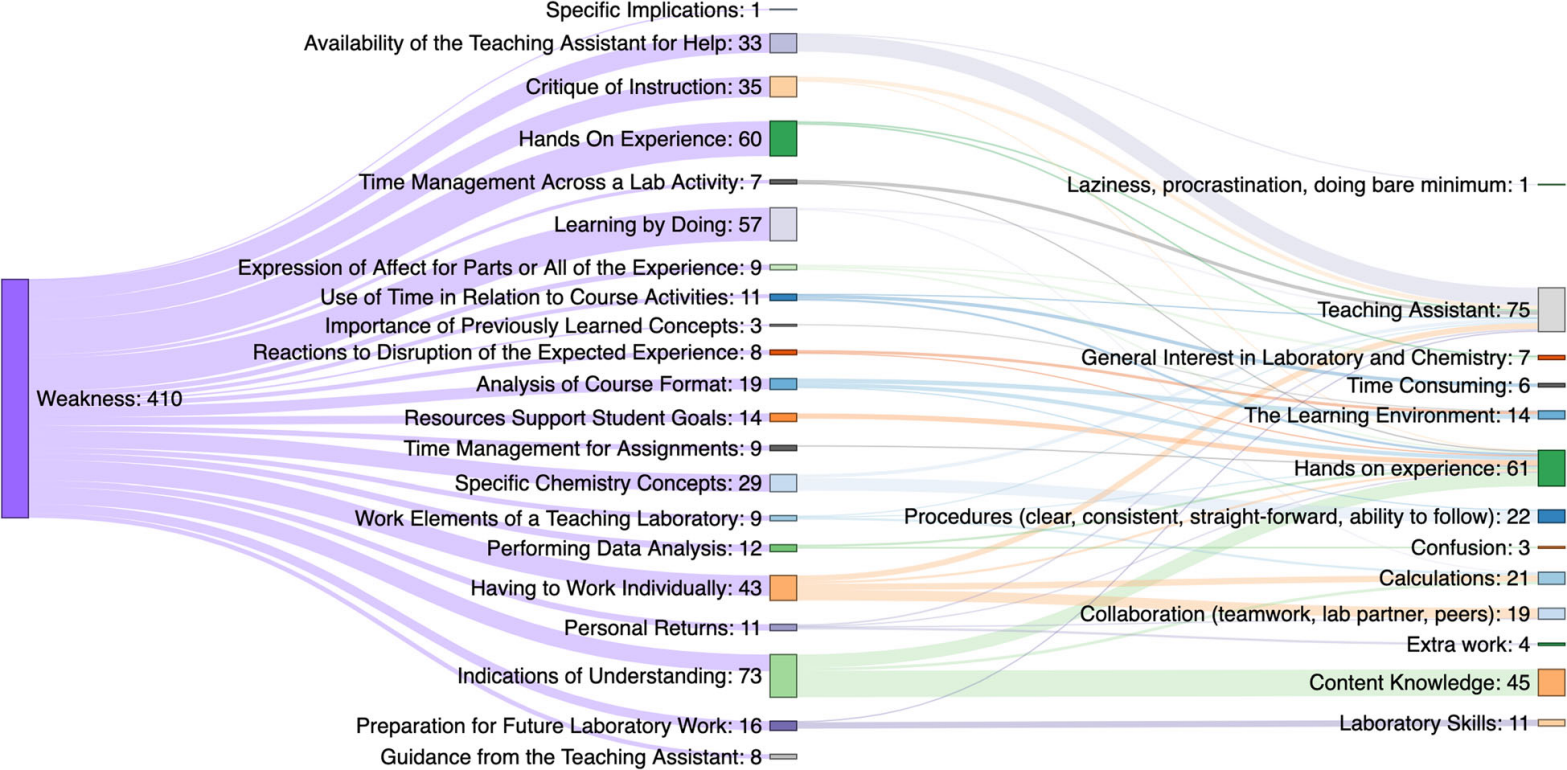
Topic and dimensions for responses from URM participants regarding weaknesses

Subsequent reviews focused on identifying similar and contrasting conceptions across each prompt. Once dimensions were created, the data was reviewed again to ensure consistency and the process was repeated until the names, definitions and dimensions adequately reflected the participants’ varied conceptions. Subgrouping by demographics and the corresponding description of results were constructed only after all data coding was complete. The outcome space, illustrated best by Fig. 4, was constructed to illuminate the association between the topics and dimensions as the range of experiences (Marton & Booth, 1997). Note that Figs. 2 & 3 do not present topic names in a parallel order because the list is prioritized based upon the occurrence by prompt. While outcome spaces in phenomenographic research can be arranged by explanatory power or even chronologically, the various topics and dimensions in the present study are illustrated hierarchically to appropriately distinguish the variety of topics and dimensions. Thus, the outcome space establishes consubstantiality between the various dimensions despite inherent differences.

## Results

The results of the sequential mixed methods analysis are presented in Table 4 and Figs. 2, 3 and 4. Results are organized according to strengths, weaknesses, positive, and negative implications across the 21 topics with dimensions (included here in the narrative with parenthesis). Occurrences of each topic are cataloged both as an enumeration and as a percentage of the total. Results are supported by student responses as quotations, where the date, record number and nature of the prompt are indicated in parentheses. For example, “Being able to watch videos to see the procedure.” (3/27, 245, Strength) indicates that this response was provided on 3/27/2020, it is identified in our dataset as record #245, and resulted from the prompt for the participant’s view about the strengths of that week’s experience.

**Table 4:** Overview of topics

Number	Name	Definition	Occurrences
1	Learning by Doing	The effect of hands on learning for the laboratory experience.	1235 (17.2%)
2	Time Management Across a Lab Activity	How students utilize time flexibly during a lab activity to maintain success.	616 (8.6%)
3	Hands On Experience	How lack of hands on experience impacts student-lab engagement.	612 (8.5%)
4	Critique of Instruction	Analysis of procedures, comments on the video, including how the two compliment or support each other	429 (6.0%)
5	Specific Chemistry Concepts	Reaction to drawing Lewis structures and then building physical models of these molecules from everyday materials.	414 (5.8%)
6	Indications of Understanding	The feelings associated with overcoming difficult laboratory elements, mainly due to poor understanding of materials.	407 (5.7%)
7	Having to Work Individually	Having to work individually as well as the transition from the prior experience of working with a lab partner.	394 (5.5%)
8	Analysis of Course Format	Views about the implementation of the specific laboratory curriculum design features.	388 (5.4%)
9	Resources Support Student Goals	How the availability of resources, including being able to watch someone do the experiment accurately or the actual hands-on experience itself allowed them to be used as needed.	358 (5.0%)
10	Specific Implications	Indications of specific implications including the most common statement that there were no implications perceived.	286 (4.0)
11	Preparation for Future Laboratory Work	Feelings about preparedness for future laboratory work in relation to other courses or for their career intentions.	270 (3.8%)
12	Availability of the TA for Help	The availability of TAs to ask questions and problem-solve.	244 (3.4%)
13	Use of Time in Relation to Course Activities	Perceptions of time spent within general activities across the course.	233 (3.2%)
14	Importance of Previously Learned Concepts	The ways that the online laboratory was perceived in relation to previously learned chemistry concepts in a related context.	223 (3.1%)
15	Time Management for Assignments	How the use of time and self-discipline affected their work in the course.	211 (2.9%)
16	Work Elements of a Teaching Laboratory	Interplay of physical and cognitive elements of teaching laboratories that are perceived as contributing to the success of student experiences .	190 (2.6%)
17	Performing Data Analysis	Important elements that make up a successful laboratory experiment.	167 (2.3%)
18	Personal Returns	Academic and emotional outputs following completion of the online laboratory.	160 (2.2%)
19	Expression of Affect for Parts or All of the Experience	Feelings derived from specific activities or the general experience with online laboratory work.	150 (2.1%)
20	Reactions to Disruption of the Expected Experience	Recognition of the underlying cause for why they are having the current experience.	109 (1.5%)
21	Guidance from the TA	Recognition of the TA as the key figure for guidance.	86 (1.2%)

A collection of selected topics, which are presented in Table 5 as examples, provide detail into the parameters of each, illustrative participant quotes for types of prompts, and how the results from the topic modeling process was used to identify the topics as categories of experience. Participant responses are included as evidence for the range of experiences that are encapsulated by the LDA derived topic.

**Table 5:** Topics and examples of participant responses with coded dimensions

Topic	Name	Definition	Example Participant Responses with Coded Dimensions
1	Learning By Doing	The effect of hands-on learning	“I could do everything at home on my own.” (4/2, 924, Strength, Accessibility (provided data, extra info, organization, do at home, replay videos)) “I think that I am able to better work with online labs after this experience because I am getting used to it.” (4/10, 8040, Positive Implication, Comfort (choice of workspace, online/home learning eases reinforcement of material)) “Since we cannot perform the labs ourselves, we cannot get familiar with using the equipment and tools. (3/27, 456, Weakness, Hands-on Experience)
7	Having to Work Individually	Having to work individually and transitioning from having a lab partner	“I think that the online sample calculations were very useful and helpful for me to figure out how to solve problems on my own without the help of my peers during lab.” (4/10, 1483, Strength, Accessibility (provided data, extra info, organization, do at home, replay videos)) “One of the weaknesses of the online experience is the lack of in-person interaction with our lab partners, our TAs, and our professors. I think that working in the lab and being able to figure out how to use each piece of equipment and doing calculations with the option for immediate assistance is one of the benefits of the lab in person.” (3/27, 4851, Weakness, Hands-On Experience) “I may forget the information faster because it is like watching a lecture, instead of teaching/performing it like we would do in the lab.” (3/27, 128, Negative Implication, Content Knowledge)
11	Preparation for Future Laboratory Work	Feelings about preparedness in relation to other courses or career	“I am becoming more familiar with how to utilize an online platform, and this will certainly be beneficial in the future as the world continues to grow and progress in terms of technology.” (4/3, 2216, Positive Implication, Technology and Tech Skills) “I can not further the laboratory skills that I may need for more advanced lab classes.” (4/10, 4034, Weakness, Laboratory Skills) “Since I have to work in the lab more in the future, I could see myself not being used to certain procedures and safety regulations while completing the lab online instead in the lab.” (3/27, 113, Negative Implication, Real Lab Experience)
18	Personal Returns	Academic and emotional outputs following completion of the online laboratory	“I’ve become more accustom to online learning, and less reliant on my TA.” (3/27, 802, Positive Implication, Independence and Self-Reliance) “While doing the post lab analysis I felt as if it was just busy work to try and keep us engaged and had very little motivation to complete it because I had not actually gone through the procedures of the lab, and I was just observing prepared videos.” (3/27, 4401, Weakness, Extra Work) “It is less engaging because you are not doing the labs yourself. You basically only get to do the dirty work: the lab report. It is fun to actually be able to do the experiment and then it is sometimes easier to write about it.” (4/2, 5646, Weakness, Hands-on Experience)

To fulfill our phenomenographic goal of capturing the most comprehensive variation in undergraduate student experience, the results are presented as individual case descriptions by group and URM subgroup. A strength of phenomenographic research is the ability to empirically study the variation in experience by assembling a hierarchy of perceptions (Marton & Pang, 2008; Marton, 1981, 1986, 1992). Our results mirror a standard phenomenography’s ontological non-dualistic perspective, and a topic occurrence hierarchy emerged. While topic occurrence can be presented in a variety of ways, Sankey diagrams of the response from all students and then URM students as a critical sub-group show interrelatedness and important differences that should be examined (Figs. 2, 3 and 4). Focusing on the experience of URM students reflects the reality of broadening inequalities with the forced digital environment due to the host of issues escalated by the pandemic.

### All participants

The sentiments expressed by all students through the topic responses overlap in terms of strengths, positive implications, weaknesses, and negative implications. However, recognizing certain patterns regarding how responses were cataloged amongst the 21 topics in Fig. 2 warrant further discussion.

Reported strengths tie predominantly to the topics of Time Management Across a Lab Activity (adaptable and flexible, independence and self-reliance) and Critique of Instruction (procedures, videos). In addition, Learning by Doing (provided information, can do at home, replay videos) and Resources Support Student Goal (provided data and tables, sample calculations, extra information) are reported strengths with comparative frequency. Positive implications show similar patterns, with a high frequency of responses clustering in the topics of Time Management Across a Lab Activity (adaptable and flexible, independence and self-reliance) and Learning by Doing (provided information, can do at home, replay videos). Notably, Time Management Across a Lab Activity was the one topic that was almost entirely made of a combination of strengths and positive implications.

Student perceptions of weakness did not striate into one or two topics like with the sentiments that elicit positive emotions. Regardless, Having to Work Individually (collaboration, calculations), Hands On Experience (hands on, content knowledge, grades), Critique of Instruction (procedures, videos), and Learning by Doing (provided information, can do at home, replay videos) are noticeably favored as a reported weakness of the laboratory by students. Learning by Doing also is the most reported negative implication as well, with Hands of Experience (hands on, procedures, content knowledge), Preparation of Future Laboratory Work (hands-on, feelings of being prepared, laboratory skills), and Specific Implications (hands on, content knowledge, grades) as additional highly reported negative implications, but to a lesser extent. Hands On Experience was interestingly the only topic made up nearly entirely of weaknesses and negative implications. Learning by Doing as a topic was shared as a near four-way tie between the strengths (to a slightly lesser extent), positive implications, weaknesses, and negative implications, making it the closest to a true neutral topic amongst the list.

### URM participants

When disaggregated, there was little difference in the sentiments expressed by URM students compared to their peers (Fig. 3). Critique of Instruction and Time Management Across a Lab Activity were the most reported strengths, with Learning by Doing and Resources Support Student Goals reported at a slightly lower rate. The following student response exemplifies the topic of Resources Support Student Goals: “Being able to watch videos to see the procedure (3/27, 245, Strength)”. When viewing positive implications, Time Management Across a Lab Activity and Learning by Doing were the most reported topics, where “Learned to teach myself with limited instructions” (4/3, 2025, Positive Implication) was a representative student response of the latter. Importantly, Time Management Across a Lab Activity is represented almost entirely by strengths and positive implications.

Regarding weaknesses reported by the URM population, Hands On Experience, Learning by Doing, Having to Work Individually, and Critique of Instruction are the most highly recognized topics by students. An example of a student-reported weakness was: “I just wish I was able to do it in person and get the in-person lab experience (4/10, 5189, Weakness)”. Negative implications have a variety of moderately reported topics such as Preparation of Future Laboratory Work, Specific Implications, and Hands on Experience, with Learning by Doing as the most reported negative implication. A student’s response regarding this sentiment was: “not being physically able to do it hinders the memorization of it” (4/10, 166, Negative Implication)” in reference to the absence of in-person sessions. Hands On Experience remained almost entirely populated by sentiments of weaknesses and negative implications. Additionally, Learning by Doing was expressed as a neutral sentiment not unlike what was reported in the All Participants category.

A brief analysis of non-URM students provided some insights. In contrast to URM, non-URM students’ responsiveness increases over time towards strengths, weaknesses, positive implications and negative implications. Concerning the topics themselves, non-URM individuals identified Critique of Instruction (procedures, videos) as the most valuable (> 46%). As indicated by participant #196, “The videos help demonstrate what the students are supposed to do. It is a quicker way to do the lab.”(3/27, 196, Strength).

When comparing the nuances of aggregation amongst the topics, there exists almost no differences in how topics were perceived between the general student population sample and the URM sample. The difference in perception between the samples did not exceed about 1% for any of the 21 topics. Given the potential for escalating inequalities during the pandemic and our general goal of broadening access and participation, understanding the needs of URM participants and attending to their views of the weaknesses is a priority. Accordingly, Fig. 4 illustrates the entire outcome space for the weaknesses indicated by URM students where the topics are further associated with the coded dimensions of the experience. This representation indicates how, for example, the loss of the Hands on Experience, as a dimension, functioned as an underlying learning issue across a number of topics, such as Indications of Understanding and Resources to Support Student Goals. Or how the topic of Availability of the Teaching Assistant for Help was largely about the lack of the person, which is the implication from the large connection to the Teaching Assistant dimension. Finally, we see that the weakness expressed in the topic of Indications of Understanding is largely a function of Content Knowledge, as indicated by the large connection to that dimension of the experience.

## Discussion

This study indicates a need for more fully clarifying student experience with online chemistry laboratory education. Especially since the existing research has largely focused on evaluating learning outcomes by making comparisons to a physical laboratory under the assumption that the two are and can be equivalent (Brinson, 2015, 2017). While we have identified 21 distinct ways that students perceived their experience during the mandatory transition to online chemistry laboratory education, Learning by Doing (17.2%), Time Management Across a Lab Activity (8.6%), Hands On Experience (8.5%), and Critique of Instruction (6.0%) were the most reported by all students.

Learning by Doing (provided information, can do at home, replay videos) is the most reported topic by all students by a large margin (> 8%). This metric is arguably the most predictable outcome, and is consistent with U. S. national results where the transition to online learning due to the pandemic was generally perceived as negative due to a loss in value across a number of dimensions, which included interest, effort and well-being (Garris & Fleck, 2020). While this devaluation in the context of a pandemic can be mitigated to an extent, physically engaging with your experiment and peers is clearly an irreplaceable tactual element that was regrettably absent.

Hands On Experience (hands on, procedures, content knowledge) is the only preeminently reported-on topic perceived as a significantly negative implication following the forced online transition. Research has shown that laboratory education not subjected to a mandatory online adaptation have mainly functioned as supplements to in-person lessons (Rowe et al., 2017) where students within these modules are allowed to acclimate to scientific practices before performing experiments, with the expectation of hands-on experience later on. Removing this expectation through a full online transition yielded an overwhelmingly negative sentiment. However, a positive take away from these findings is how seemingly simply the sentiment could be mitigated with more preparation. It also speaks to the value that students find or anticipate in face-to-face laboratory experiences, a sentiment that seems to be missing or disregarded in the existing research that assumes that a virtual version provides a more accessible emulation of a physical alternative (Reeves & Crippen, 2020).

The high rate of occurrence for the topics of Time Management Across a Lab Activity (adaptable and flexible, independence and self-reliance) and Critique of Instruction (procedures, videos) likely has a strong connection to the context of the pandemic. The definition and dimension of these topics align well with the reported host of issues that have been escalated by the pandemic, including the broadening of inequalities that come with dragooning students into a fully digital experience (Bolaños & Salinas, 2021; Engelbrecht et al., 2020). The rushing of curriculum design and the nature of an online laboratory course environment also made educators and teaching assistants less accessible, resigning students to their own devices more often and thus not managing time as efficiently (Kamanetz, 2020). The findings for both URM and non-URM students alike are generally consistent with other emerging reports that tend to use closed-ended items, which assume that the variation of experience is known in advance (Hsu & Rowland-Goldsmith, 2021).

While delineating 21 topics in detail is beyond the scope of any one paper, collecting data on a forced in-person/online (hybrid) science laboratory course yielded some additional findings worth mentioning. For example, the topic Personal Returns (2.1% total response occurrence) measures the academic and emotional outputs following the completion of the online laboratory experience. Contributing factors may include the oversupply of information given to students to replace the wealth of haptic feedback in a physical laboratory, the careful leveraging of resources to maximize student success, as well as a heavier emphasis on improving students’ professional skills (lab-related skills achievable remotely).

Regardless of having a low total response occurrence (3.4%), the topic Availability of the TA for Help was reported as the foremost positive implication for all examined student metrics. Hands-on instruction does not scale effectively into a lecture setup, and TA's positive impact as individual student guides was even more necessary and thankfully recognized by students.

Undergraduate science students are looking for experiences that go beyond the physical laboratory (Deacon & Hajek, 2011; Hsu & Rowland-Goldsmith, 2021) and online learning does not have to be a simple emulation of what is experienced on campus. For instance, participants perceived the isolation resulting from the loss of a laboratory partner. While a more isolated experience can be important in some instances, it is not indicative of the collaborative nature of science practice (Wuchty et al., 2007). Further investigation can inform us about what they perceive as missing or the different ways that they use the content of a course in their degree programs.

The results revealed a varied perspective regarding online learning with students identifying different components that they perceived as hindering or enhancing their experience. This supports our belief that computer mediated STEM learning experiences should be designed to support a varied learner perspective. Employing a User Experience (UX) design approach would aid in designing for a broader audience by using design tools such as data-driven personas and scenarios to capture the goals, values, needs, and actions within the identified user-group (Minichiello et al., 2018).

The results have particular relevance for those interested in student learning, including the conceptual changes that are occurring at the postsecondary level. For example, the topic of Expression of Affect for Parts or All of the Experience indicates that students were enthusiastic about attending and partaking in chemistry laboratory before the transition and that their negative feelings were largely due to the abrupt nature of the transition to online. This suggests that students find value in physical laboratory education, a counter narrative to the promotion of online laboratory education as an equivalent alternative. Also notable is the topic of Importance of Previously Learned Concepts, which suggests that students recognize and appreciate the utility of prior knowledge and its significance for success in the learning process. Delineating and enhancing these notions offer great potential for improving learning in a more targeted manner.

The COVID-19 Pandemic has pushed teachers, students, education specialists, and researchers to adapt to rapid technology implementations at an unprecedented level. Exploring the sentiments expressed by the student population during a forced online transition during a pandemic is imperative for determining changes in motivation, time management, and communication strategies. Laboratory education from a rapid and forced transition in modality combined with a lack of traditional hands-on activities created a unique scenario for students and educators that has not been covered in modern literature. Research of pandemic-centered learning environments puts into perspective what students need to help them learn.

Critical directions for future research in this genre includes the promotion of collaborative student efforts, how the nature of science in an online setting can be understood, and the management of barriers to implementation of online elements. Due to the diverse nature of our student participants, including those that may never have considered taking an online course, these results may also serve as a needs assessment for a future expansion of online laboratory education.

### Implications and future of the course

Since Spring 2020, the course has reverted to a principally on-campus and face-to-face offering, but it now includes a number of online enhancements as part of a blended or hybrid learning approach. For example, the results indicated a distinct need for improved peer-to-peer and student-to-TA interactions. Accordingly, a group of previously successful undergraduate students (approximately four per semester) now host an online discussion board where they respond to questions about each week’s activities. This response system has proven to be incredibly popular with enrolled students. Online tasks now involve breakout room discussions, both within a single room as well as across a group of rooms where student pairs interact, compare and discuss variation in results and possible sources of error. There is a clear indication that this modification has resulted in improved student interaction. TA training has become more of a priority, especially as it relates to developing social relationships, using videoconferencing tools effectively and understanding the needs and experience from the perspective of students. Instructors note that TAs are spending more time talking to students instead of just watching them and only jumping in if they anticipate a need for help or answering a raised hand.

The infrastructure created as part of the mandatory online course offering and perspective provided by the results of this study has made it possible to offer online make-up activities that are flexible to the needs of roughly 1100 students who continue to require them due to COVID exposure, quarantine, or other form of illness (e.g., positive COVID-19 test, Strep, flu, etc.). Students are permitted to use these sessions for up to two approved/excused absences. Video-based instruction is incorporated throughout the online course materials, where rather than being given a list of materials and equipment, students are provided with a video of equipment and materials and are required to make their own list. For lengthy procedures, a combination of hands-on and video activities are used. Video instruction of how to use equipment is used liberally to better prepare students prior to formal activities for such topics as how to use a Bunsen burner or how to dispose of waste properly, etc. All videos are curated and continuously updated, to include audio, annotations, and closed captioning so as to improve accessibility.

### Limitations

A few limitations should be considered when interpreting the results, the most pertinent being the use of self-report data. Due to the circumstances outside of the classroom in a global pandemic, the participant responses may have some amount of misattribution or bias. The participants may be using the surveys, which were intended to collect data about the chemistry laboratory education experience specifically, as a forum to voice their frustrations that may have been rooted outside of the laboratory education context. Another possible limitation is the quality and/or length of the responses that were collected from the students. Due to the nature of both the survey questions and the demographics of the participants, some of the collected responses were either short in length, vague in wording or both (e.g., “none” or “n/a”). Though our use of Gibbs sampling in the topic modeling process was intended to address this issue, such responses may have lacked sufficient length and specificity. It is possible that these responses created some amount of signal noise that affected the generation of the topics.

## Conclusion

Though online learning was an established and viable form of education before the pandemic, the mandatory transition to requiring this for everyone, particularly in the context of university laboratory education, challenged our capacities, assumptions and the boundaries of our collective knowledge and understanding. Aside from the tremendous negative implications due to our lack of preparation and capacity, this phenomenon presented an unparalleled opportunity to more fully understand the potential of this technological application at the most diverse and grandest of scales. This study reminds us that the student experience must be the primary consideration for any educational endeavor and needs to continue as a principal point of emphasis for research and development. As the greater our understanding for the variation in experience, the better our capacity for providing the experience and achieving the outcomes that we desire.

## Data Availability

The datasets used and/or analyzed during the current study are available from the corresponding author on reasonable request.

## Abbreviations

COVID-19: Coronavirus Disease 2019
ICT: Information and Computer Technology
LDA: Latent Dirichlet Allocation
NRC: National Research Council
STEM: Science, technology, engineering, mathematics
TA: Teaching Assistant
URM: Underrepresented Ethnic Minority
U. S.: United States of America
UX: User Experience

## References

[CR1] Ashworth, P., & Lucas, U. (2000). Achieving empathy and engagement: A practical approach to the design, conduct and reporting of phenomenographic research. *Studies in Higher Education*, *25*10.1080/713696153, 3, 295, 308

[CR2] Blei, D. M., & Lafferty, J. D. (2007). A correlated topic model of science. *The Annals of Applied Statistics*, *1*(1), 17–35. 10.1214/07-AOAS114.

[CR3] Boda, P. A. (2019). The conceptual and disciplinary segregation of disability: a phenomenography of science education graduate student learning. *Research in Science Education*, 1–34. 10.1007/s11165-019-9828-x.

[CR4] Bolaños, F., & Salinas, Á. (2021). Secondary vocational education students’ expressed experiences of and approaches to information interaction activities within digital environments: A Phenomenographic study. *Education and Information Technologies*, *26*(2), 1955, 1975. 10.1007/s10639-020-10322-0.

[CR5] Brenner, K., Dahlberg, M. L., & Alper, J. (2021). Undergraduate and Graduate STEM Students’ Experiences During COVID-19. In K. Brenner, M. L. Dahlberg, & J. Alper (Eds.), *Proceedings of a Virtual Workshop Series*. National Academies Press. 10.17226/26024.

[CR6] Brinson, J. R. (2015). Learning outcome achievement in non-traditional (virtual and remote) versus traditional (hands-on) laboratories: A review of the empirical research. *Computers & Education*, *87*, 218–237. 10.1016/j.compedu.2015.07.003.

[CR7] Brinson, J. R. (2017). A further characterization of empirical research related to learning outcome achievement in remote and virtual science labs. *Journal of Science Education and Technology*, *26*(5), 546–560. 10.1007/s10956-017-9699-8.

[CR8] Crawford, B. A. (2007). Learning to teach science as inquiry in the rough and tumble of practice. *Journal of Research in Science Teaching*, *44*(4), 613–642. 10.1002/tea.20157.

[CR9] Creswell, J. W., & Clark, V. L. P. (2017). *Designing And Conducting Mixed Methods Research*, (p. 520). Los Angeles: Sage Publications.

[CR10] de Jong, T., Linn, M. C., & Zacharia, Z. C. (2013). Physical and virtual laboratories in science and engineering education. *Science*, *340*(6130), 305–308. 10.1126/science.1230579. 23599479 10.1126/science.1230579

[CR11] Deacon, C., & Hajek, A. (2011). Student perceptions of the value of physics laboratories. *International Journal of Science Education*, *33*(7), 943–977. 10.1080/09500693.2010.481682.

[CR12] Engelbrecht, J., Borba, M. C., Llinares, S., & Kaiser, G. (2020). Will 2020 be remembered as the year in which education was changed? ZDM : The International Journal on Mathematics Education, 1–4. 10.1007/s11858-020-01185-3, 2020 10.1007/s11858-020-01185-3 PMC7374655 32837580

[CR13] Feldon, D. F., & Tofel-Grehl, C. (2018). Phenomenography as a foundation for mixed models research. *American Behavioral Scientist*, *62*(7), 887–899. 10.1177/0002764218772640.

[CR14] Fraillon, J., Ainley, J., Schulz, W., Friedman, T., & Gebhardt, E. (2014). *Preparing for Life in a Digital Age*. Springer International Publishing. 10.1007/978-3-319-14222-7.

[CR15] Garris, C. P., & Fleck, B. (2020). Student evaluations of transitioned-online courses during the COVID-19 pandemic. *Scholarship of Teaching and Learning in Psychology*. 10.1037/stl0000229.

[CR16] Gewin, V. (2020). Five tips for moving teaching online as COVID-19 takes hold. *Nature*, *580*(7802), 295–296. 10.1038/d41586-020-00896-7. 32210377 10.1038/d41586-020-00896-7

[CR17] Gott, R., & Duggan, S. (1996). Practical work: Its role in the understanding of evidence in science. *International Journal of Science Education*, *18*(7), 791–806. 10.1080/0950069960180705.

[CR18] Han, F., & Ellis, R. A. (2019). Using phenomenography to tackle key challenges in science education. *Frontiers in Psychology*, *10*10.3389/fpsyg.2019.01414, 1414. 31293478 10.3389/fpsyg.2019.01414 PMC6603223

[CR19] Hsu, J. L., & Rowland-Goldsmith, M. (2021). Student perceptions of an inquiry-based molecular biology lecture and lab following a mid-semester transition to online teaching. *Biochemistry and Molecular Biology Education*, *49*(1), 15–25. 10.1002/bmb.21478. 33301654 10.1002/bmb.21478

[CR20] Kalyuga, S. (2007). Expertise reversal effect and its implications for learner-tailored instruction. *Educational Psychology Review*, *19*(4), 509–539. 10.1007/s10648-007-9054-3.

[CR21] Kamanetz, A. (2020). *Panic-gogy’: Teaching online classes during the coronavirus pandemic. NPR Special Series: The Coronavirus Crisis*.

[CR22] König, J., Jäger-Biela, D. J., & Glutsch, N. (2020). Adapting to online teaching during COVID-19 school closure: teacher education and teacher competence effects among early career teachers in Germany. *European Journal of Teacher Education*, 1–15. 10.1080/02619768.2020.1809650.

[CR23] Kotu, V., & Deshpande, B. (2015). *Predictive Analytics and Data Mining: Concepts and Practice with Rapidminer*. Morgan Kaufmann.

[CR24] Linn, M. C., & Eylon, B.-S. (2011). *Science learning and instruction: taking advantage of technology to promote knowledge integration*. Routledge. 10.4324/9780203806524.

[CR25] Lunnetta, V. N., Hofstein, A., & Clough, M. P. (2007). Learning and teaching in the school science laboratory: An analysis of research, theory, and practice. In S. K. Abell, & N. G. Lederman (Eds.), *Handbook of Research on Science Education*, (pp. 393–442). New York: Lawrence Earlbaum.

[CR26] Ma, J., & Nickerson, J. V. (2006). Hands-on, simulated and remote laboratories: A comparative literature review. *ACM Computing Surveys*, *38*(3), 1–24. 10.1145/1132960.1132961.

[CR27] Makransky, G., Thisgaard, M. W., & Gadegaard, H. (2016). Virtual simulations as preparation for lab exercises: Assessing learning of key laboratory skills in microbiology and improvement of essential non-cognitive skills. *PLoS One*, *11*(6), e0155895. 10.1371/journal.pone.0155895. 27253395 10.1371/journal.pone.0155895 PMC4890735

[CR28] Marton, F. (1981). Phenomenography ? Describing conceptions of the world around us. *Instructional Science*, *10*(2), 177–200. 10.1007/BF00132516.

[CR29] Marton, F. (1986). Phenomenography—{a} {research} {approach} to {investigating} {different} {understandings} of {reality}. *Journal of Thought*, *21*(3), 28–49 http://www.jstor.org/stable/42589189.

[CR30] Marton, F. (1992). Phenomenography and “the art of teaching all things to all men”. *International Journal of Qualitative Studies in Education*, *5*(3), 253–267. 10.1080/0951839920050305.

[CR31] Marton, F., & Booth, S. (1997). *Learning and Awareness (Educational Psychology Series)*, (p. 240). New York: Routledge.

[CR32] Marton, F., & Booth, S. (2013). *Learning and awareness*. New York: Routledge.

[CR33] Marton, F., & Pang, M. F. (2008). The idea of phenomenography and the pedagogy of conceptual change. *International Handbook of Research on Conceptual Change*, (1st ed., pp. 533–559). Routledge.

[CR34] Means, B., Neisler, J., & Langer Research Associates. (2020). Suddenly Online: A NationalSurvey of Undergraduates During the COVID-19 Pandemic. Digital Promise. Retrieved April 13, 2021 from https://digitalpromise.org/wp-content/uploads/2020/07/ELE_CoBrand_DP_FINAL_3.pdf.

[CR35] Merchant, Z., Goetz, E. T., Keeney-Kennicutt, W., Kwok, O., Cifuentes, L., & Davis, T. J. (2012). The learner characteristics, features of desktop 3D virtual reality environments, and college chemistry instruction: A structural equation modeling analysis. *Computers & Education*, *59*(2), 551–568. 10.1016/j.compedu.2012.02.004.

[CR36] Millar, R. (2004). The role of practical work in the teaching and learning of science (Commissioned Paper No. 308). Committee on High School Science Laboratories: Role and Vision. *National Academy of Sciences* https://sites.nationalacademies.org/cs/groups/dbassesite/documents/webpage/dbasse_073330.pdf.

[CR37] Minichiello, A., Hood, J. R., & Harkness, D. S. (2018). Bringing user experience design to bear on STEM education: A narrative literature review. *Journal for STEM Education Research*, *1*(1-2), 7–33. 10.1007/s41979-018-0005-3.

[CR38] National Research Council [NRC] (2006). *America’s lab report: Investigations in high school science*. Washington, DC: National Academies Press.

[CR39] Newton, G., & Martin, E. (2013). Blooming, SOLO taxonomy, and phenomenography as assessment strategies in undergraduate science education. *Journal of College Science Teaching*, *43*(2), 78. http://www.jstor.org/stable/43631075. 10.2505/4/jcst13_043_02_78.

[CR40] Nikolenko, S. I., Koltcov, S., & Koltsova, O. (2017). Topic modelling for qualitative studies. *Journal of Information Science*, *43*. 10.1177/0165551515617393.

[CR41] Nolen, S. B., & Koretsky, M. D. (2018). Affordances of virtual and physical laboratory projects for instructional design: Impacts on student engagement. *IEEE Transactions on Education*, *61*(3), 226–233. 10.1109/TE.2018.2791445.

[CR42] Potkonjak, V., Gardner, M., Callaghan, V., Mattila, P., Guetl, C., Petrović, V. M., & Jovanović, K. (2016). Virtual laboratories for education in science, technology, and engineering: A review. *Computers & Education*, *95*, 309–327. 10.1016/j.compedu.2016.02.002.

[CR43] Reeves, S. M., & Crippen, K. J. (2020). Virtual laboratories in undergraduate science and engineering courses: a systematic review, 2009–2019. *Journal of Science Education and Technology*, *30*(1), 16–30. 10.1007/s10956-020-09866-0.

[CR44] Reid, N., & Shah, I. (2007). The role of laboratory work in university chemistry. *Chemical Education Research and Practice*, *8*, 2, 172, 185, 10.1039/B5RP90026C

[CR45] Rowe, R. J., Koban, L., Davidoff, A. J., & Thompson, K. H. (2017). Efficacy of online laboratory science courses. *Journal of Formative Design in Learning*, *2*(1), 56–67. 10.1007/s41686-017-0014-0.

[CR46] Salomatin, K., Yang, Y., & Lad, A. (2009). Multi-field Correlated Topic Modeling. In C. Apte, H. Park, K. Wang, & M. J. Zaki (Eds.), *Proceedings of the 2009 SIAM International Conference on Data Mining*, (pp. 628–637). Society for Industrial and Applied Mathematics. 10.1137/1.9781611972795.54.

[CR47] Sbalchiero, S., & Eder, M. (2020). Topic modeling, long texts and the best number of topics. Some Problems and solutions. *Quality & Quantity*. 10.1007/s11135-020-00976-w.

[CR48] Simonsmeier, B. A., Flaig, M., Deiglmayr, A., Schalk, L., & Schneider, M. (2021). Domain-specific prior knowledge and learning: A meta-analysis. *Educational Psychologist*, *57*(1), 31–54. 10.1080/00461520.2021.1939700.

[CR49] Thompson, P. (2013). The digital natives as learners: Technology use patterns and approaches to learning. *Computers & Education*, *65*, 12–33. 10.1016/j.compedu.2012.12.022.

[CR50] Tight, M. (2018). *Higher Education Research* (1st ed., p. 576). Bloomsbury UK.

[CR51] Wan, T., Geraets, A. A., Doty, C. M., Saitta, E. K. H., & Chini, J. J. (2020). Characterizing science graduate teaching assistants’ instructional practices in reformed laboratories and tutorials. *International Journal of STEM Education*, *7*10.1186/s40594-020-00229-0, 1, 30

[CR52] Wang, C.-Y., Wu, H.-K., Lee, S. W.-Y., Hwang, F.-K., Chang, H.-Y., Wu, Y.-T., … Lin, J.-W. (2014). A review of research on technology-assisted school science laboratories. *Journal of Educational Technology & Society*, *17*(2), 307–320.

[CR53] Wuchty, S., Jones, B. F., & Uzzi, B. (2007). The increasing dominance of teams in production of knowledge. *Science*, *316*(5827), 1036–1039. 10.1126/science.1136099. 17431139 10.1126/science.1136099

[CR54] Yates, C., Partridge, H., & Bruce, C. (2012). Exploring information experiences through phenomenography. *Library and Information Research*, *36*(112), 96–119. 10.29173/lirg496.

[CR55] Yin, R. K. (2002). *Case study research: Design and methods*, (vol. 5, 3rd ed., ). Thousand Oaks: Sage.

